# Insight into ocular complications of West Nile Virus: A case report of chorioretinal scarring^☆^

**DOI:** 10.1016/j.idcr.2024.e02095

**Published:** 2024-10-15

**Authors:** Hamza Inayat, Anna Branch, James J. Armstrong, Verena Juncal, Courtney Casserly

**Affiliations:** a Schulich School of Medicine & Dentistry, Western University, London, Ontario, Canada; b Department of Ophthalmology, Schulich School of Medicine & Dentistry, Western University, London, Ontario, Canada; c Ivey Eye Institute, St. Joseph’s Hospital, London, Ontario, Canada; d Department of Clinical Neurological Sciences, Schulich School of Medicine & Dentistry, Western University, London, Ontario, Canada

**Keywords:** West Nile Virus, Chorioretinal Scar, Retina

## Abstract

**Purpose:**

This case report aims to emphasize the significance of the ocular manifestations in individuals with West Nile Virus (WNV) infection, with primary neurological involvement. By presenting a case of chorioretinal scarring secondary to WNV, we highlight the importance of a thorough ophthalmological evaluation in suspected cases of WNV to identify potential sight-threatening complications.

**Observations:**

A 63-year-old woman presented with neurological symptoms following a trip to Denver, USA, including headaches, fever, and decreased consciousness. Despite initial treatment for suspected meningoencephalitis, her condition deteriorated, leading to focal seizures and profound weakness. Ophthalmological examination revealed chorioretinal lesions consistent with WNV retinopathy.

**Conclusions:**

and Importance: WNV can present with neurological symptoms, and ocular complications can lead to significant visual impairment. This case report highlights the importance of asking individuals with suspected WNV about ocular symptoms. Despite the absence of a preventive treatment for ocular manifestations, clinicians must stay attentive to ocular symptoms in patients to mitigate potential complications, such as chorioretinal neovascularization, which can be treated with anti-vascular endothelial growth factor therapy injections.

## Introduction

West Nile Virus (WNV), a mosquito-borne arbovirus, is predominantly recognized for its neurological involvement. However, its ocular manifestations are less well recognized despite being the next most commonly affected system [1]. These ocular effects, although less frequently reported than other neurological symptoms [2], can include chorioretinitis, uveitis, retinal hemorrhages, and chorioretinal scarring [3], We present a patient presenting with chorioretinal scarring from a WNV infection, shedding light on the potentially severe ocular complications associated with this virus. This report aims to highlight the importance of looking for ocular findings in patients with suspected WNV. It necessitates a thorough ophthalmological evaluation in patients with suspected WNV to identify and manage these potentially sight-threatening complications. While neurological symptoms are often the presenting concern, ocular complications can lead to significant morbidity with long-term visual impairment.

## Case presentation

### Patient description

The patient is a 63-year-old white woman who presented with a decreased level of consciousness following a trip to Denver, Colorado from her hometown in Southwestern Ontario, Canada. Her medical history was notable only for asthma, with no known allergies. At the time of presentation, her only medications were Symbicort 200 mcg/6 mcg 1 puff daily and Ventolin as needed. Her previous ocular history consisted of laser peripheral iridotomy in both eyes ten years prior to presentation.

### Clinical presentation

The patient's symptoms began shortly after her return from Denver USA, where she had been hiking, attended a rodeo, and visited a zoo. Initial symptoms included headaches and fever. Over the course of a week, her condition worsened as she developed progressive weakness, blurry vision, and decreased level of consciousness. She also had an erythematous rash. She was initially treated with dexamethasone, ceftriaxone, vancomycin, ampicillin and acyclovir for suspected meningoencephalitis. The infectious disease service was consulted to guide investigations.

**Fig. 1:**
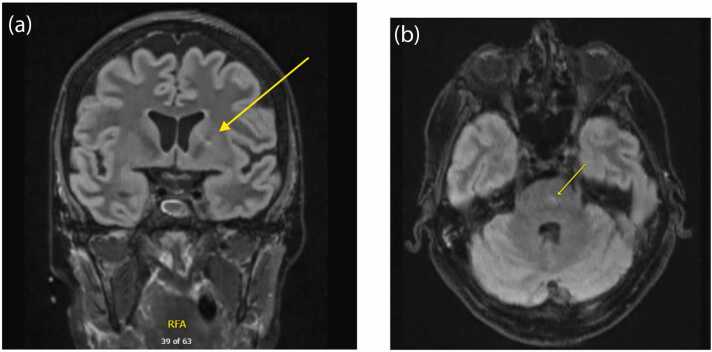
MRI head illustrating small foci of increased signal in the basal ganglia (a) and central pons (b).

Initial laboratory tests showed white blood cell count (9.7 ×10 *9/L), neutrophils (8.1 ×10 *9/L), normal liver enzymes, creatinine, lactate, and ammonia. CSF studies showed mild lymphocytic pleocytosis in cerebrospinal fluid (15 cells), a normal serum glucose of 6.8 mmol/L, and CSF glucose (5.1 mmol/L),). MRI imaging (Fig. 1) revealed small foci of increased signal in the basal ganglia and central pons. Microbiology tests, including blood, urine, and CSF cultures were negative; and viral PCR for COVID-19, HSV, VZV, and enterovirus were negative. Tests for WNV IgG and IgM (blood) and IgM (CSF) were positive, which allowed us to make the diagnosis of neuroinvasive WNV. At this stage, steroids, antibiotics and antivirals were stopped.

The patient had persistent decreased level of consciousness, with intermittent focal seizures with tonic eye deviation, and accompanying electrographic seizures. She was treated with levetiracetam for seizures. As she woke up, she was found to have profound weakness which was diagnosed on EMG as a distal axonal motor neuronopathy. She eventually underwent a tracheostomy and was weaned down on her antiepileptic drugs in the following weeks after improvement in her mental status and on EEG. She improved clinically and her seizures resolved; however, upon regaining consciousness more than a month after admission, she reported impaired vision, predominantly in the left eye. Additionally, she reported experiencing bilateral central vision loss.

### Ophthalmological examination: day 66 after admission

Visual acuity testing revealed 20/20 vision in the right eye and 20/50 in the left, and no improvement with pinhole testing. Intraocular pressures were 13/12 by iCare tonometry. She had a left relative afferent pupillary defect (RAPD).

The anterior segment examination revealed slightly shallow anterior chambers in both eyes, with no signs of a cellular reaction. Trace white blood cells were noted in the left anterior vitreous, while the right anterior vitreous appeared clear.

**Fig. 2:**
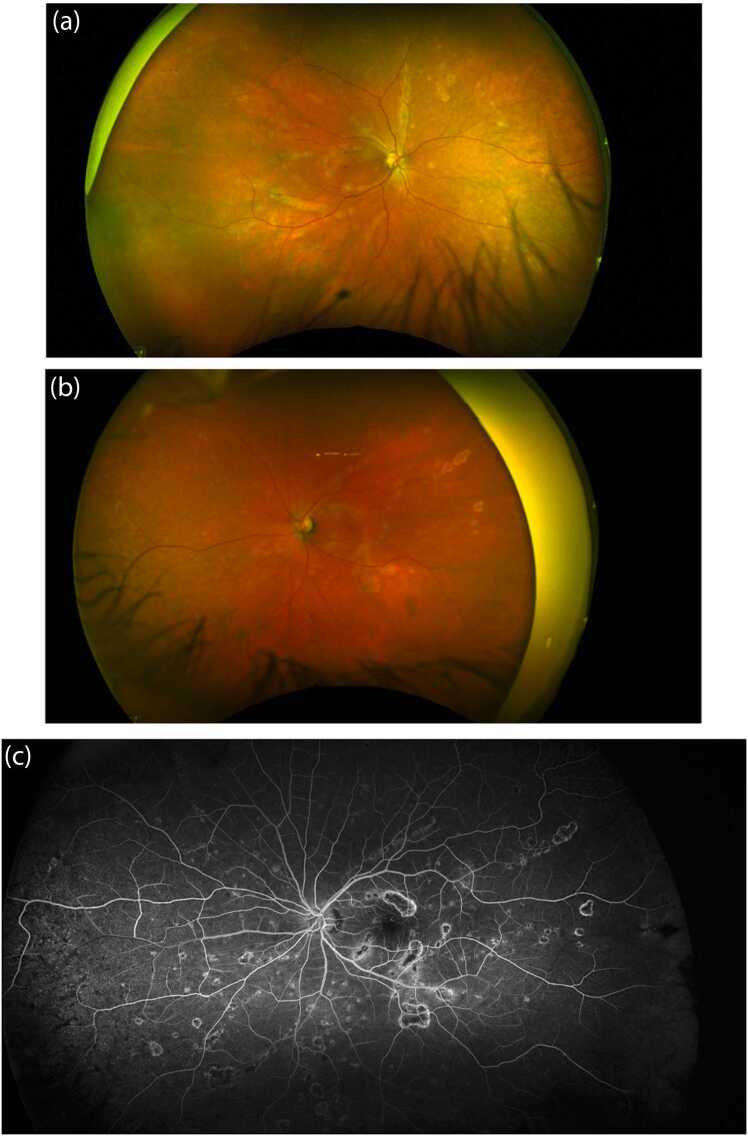
Wide-field fundus color photos and fluorescein angiography demonstrating chorioretinal scars: 2a) wide-field fundus color photo right eye 2b) wide-field fundus color photo left eye, 2c) Wide-field fluorescein angiography of the left eye demonstrating chorioretinal scarring with late staining of borders.

**Fig. 3:**
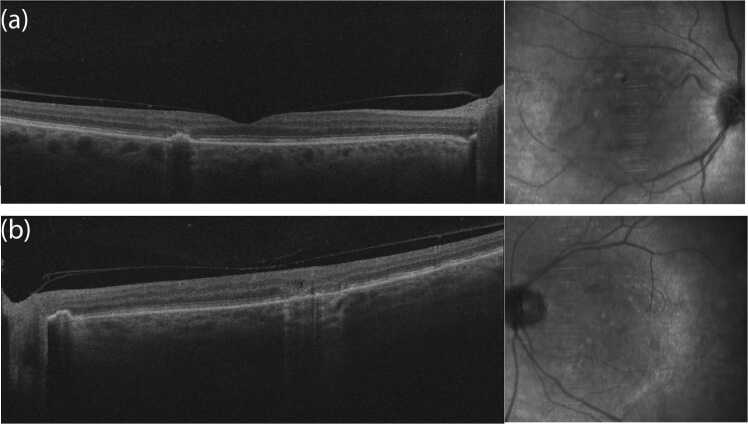
Optical coherence tomography (OCT) images demonstrating chorioretinal lesions. 3a) OCT Right eye, 3b) OCT Left eye.

On dilated fundus examination, several scattered chorioretinal lesions were observed in a linearly arranged pattern in both eyes. Most lesions were already partially pigmented. Intravenous Fluorescein Angiography (IVFA) did not demonstrate any perivascular leakage in either eye. Most lesions presented with a central hypofluorescence with peripheral hyperfluorescence. Optical Coherence Tomography (OCT) of the macula of both eyes showed perifoveal areas of outer retina disruption and focal irregular pigment epithelium detachments. Some areas of outer retinal loss had small overlying intraretinal cysts. Wide-field color photos, IVFA and OCT scans are illustrated in Fig. 2, Fig. 3 respectively.

The patient was diagnosed with West Nile retinopathy, with all retinal lesions appearing to be inactive. The patient was counseled about the importance of ongoing ocular monitoring due to increased risks of ocular complications, such as the development of choroidal neovascularization.

## Discussion

This case report of a 63-year-old woman presenting with chorioretinal scarring following a West Nile Virus (WNV) infection serves as a reminder that, while WNV is primarily known for its neurological symptoms, there are ocular complications that can lead to significant, irreversible visual dysfunction.

### Ocular manifestations of WNV

The patient's ocular findings, particularly the chorioretinal scarring, represent one manifestation of WNV infection. Belonging to the Flaviviridae family, WNV is a single-stranded RNA virus that hijacks host cell machinery to produce new viral particles using the host's own proteins. While the precise molecular mechanisms underlying WNV infection are still being explored, recent studies suggest that WNV might enter cells by interacting with toll-like receptor 3 (Tlr3) and CCR5 [4], [5]. This interaction is believed to trigger a Tlr3-mediated inflammatory response, which compromises the integrity of the blood-brain barrier. The resulting weakened barrier potentially allows the virus to bypass the blood-brain or blood-retina barrier, leading to central nervous system involvement, such as encephalitis and retinopathy [6].

It is theorized that WNV might access ocular tissues either by spreading through the bloodstream to the blood vessels of the choroid or by tracking directly from the central nervous system along the optic nerve fibers to the retina, retinal pigmented epithelium (RPE), and choroid. This second theory is supported by the observation that the arrangement of chorioretinal lesions often aligns with the anatomical layout of retinal nerve fibers, indicating a linear distribution pattern [3].

Due to the presence of blood-retinal barriers in the eye, it is considered an immune-privileged organ protected against systemic infections. These distinctive anatomical features could potentially explain the infrequent occurrence of ocular manifestations associated with WNVI in non-neuroinvasive scenarios [7].

It is important to monitor the ocular involvement seen in patients with WNV, particularly to prevent/treat for choroidal neovascularization. Currently, there is no available ocular treatment for WNV to prevent scarring. Only treatment available is anti-vascular endothelial growth factor (VEGF) therapy injections for choroidal neovascularization. Patients with a recent history of travel to WNV endemic areas who have developed encephalitis have higher rates of ocular involvement [6].

Although choroidal neovascularization as a complication of chorioretinal scarring is uncommon, it is important to perform a ophthalmologic assessment to guide diagnostic efforts. Non-ophthalmologists must recognize the need to consult ophthalmology when dealing with inflammatory neurologic conditions, especially when visual symptoms are present or when the underlying cause is unclear. Such consultations are invaluable in the diagnostic process, helping to identify prevent visual consequences from conditions like WNV, which may not be initially considered.

WNV often manifests in the eye through conditions such as chorioretinitis, optic neuritis, and vitritis. Chorioretinitis, characterized by inflammation of the choroid and retina, can cause symptoms like blurred vision, floaters, and, in severe cases, vision loss. Optic neuritis, involving inflammation of the optic nerve, may lead to a sudden reduction in vision, altered color perception, and eye pain with movement. Vitritis, the inflammation of the vitreous humor, typically presents with floaters and decreased visual acuity. Recognizing these symptoms should prompt an immediate referral to an ophthalmologist for further evaluation.

### Diagnostic challenges

Diagnosing ocular manifestations of WNV can be challenging due to the variability in presentations [8]. In this case, the patient's history of travel, coupled with the onset of encephalitis symptoms, and input from the infectious diseases team, was critical in guiding diagnostic testing. The use of specific tests for WNV, including IgG in serum and IgM in serum and CSF, were instrumental in confirming the infection. However, due to challenges of fully assessing vision in the ICU setting, it was not done until later in the disease course that ocular involvement was diagnosed. Clinicians should maintain a high index of suspicion for vision changes in patients with possible WNV. In this case, the patient’s altered level of awareness, as well as a prolonged admission to the intensive care unit, were initial barriers to more comprehensive ophthalmological assessment. The ability to performing detailed ophthalmological assessments may be limited for non-ophthalmologists, without specialized equipment, particularly in patients who are supine and/or with decreased level of awareness.

### Management and prognosis

Management of WNV-related ocular complications primarily revolves around supportive care, as there is no specific treatment for the ocular manifestations. However, monitoring of ocular involvement is important e as patients can develop neovascularization, where anti-vascular endothelial growth factor therapy may be considered to prevent further vision complication.

## Conclusion

In conclusion, this case report contributes valuable insights into the spectrum of ocular manifestations associated with WNV infection. It underscores the importance of an ophthalmologic examination particularly in those presenting with ocular symptoms or unexplained visual changes, or for conditions that could affect one’s vision. While the development of choroidal neovascularization is a rare but serious complication, recognition of the need for a thorough ophthalmologic evaluation can help with management, especially in regions where WNV is not commonly encountered. Further research is warranted to better understand the pathophysiology of WNV-associated ocular complications and to explore potential novel therapeutic strategies.

## Ethical approval

No ethics approval for case report.

## Funding/Support

None.

## Funding/Support

No funding or grant support.

## Disclaimers

None.

## Patient Consent

Consent to publish this case report has been obtained from the patient in writing.

## Consent

Written informed consent was obtained from the patient for publication of this case report and accompanying images. A copy of the written consent is available for review by the Editor-in-Chief of this journal on request.

## Authorship

All authors attest that they meet the current ICMJE criteria for Authorship.

## Author Statement

The case report is in compliance with relevant laws and institutional guidelines. Informed consent was obtained from the patient for the writing of this case report. There are no conflicts of interest from the authors.

## CRediT authorship contribution statement

**Hamza Inayat:** Writing – review & editing, Writing – original draft, Conceptualization. **Anna Branch:** Writing – review & editing, Writing – original draft, Conceptualization. **James Armstrong:** Writing – review & editing, Writing – original draft, Conceptualization. **Verena Juncal:** Writing – review & editing, Writing – original draft, Supervision, Conceptualization. **Courtney Casserly:** Writing – review & editing, Writing – original draft, Supervision, Conceptualization.

## Declaration of Competing Interest

There are no conflict of interest to disclose for the authors.
